# Modulating a summer ophthalmology research experience for medical students during the COVID-19 pandemic

**DOI:** 10.5116/ijme.600e.c0e4

**Published:** 2021-02-26

**Authors:** Timothy W. Corson, David K. Wallace

**Affiliations:** 1 Eugene and Marilyn Glick Eye Institute, Department of Ophthalmology, Indiana University School of Medicine, Indianapolis, Indiana, USA

**Keywords:** Research education, vision science, COVID-19, virtual research, summer research program

## To the Editor

There is a leaky pipeline of physician-scientists in the United States,^1^and ophthalmologist-scientists in particular.^2^^,^^3^ Although most ophthalmology residency programs view applicants with research expertise favorably, relatively few programs offer truly substantive research opportunities for their residents, primarily because their time is committed to the demands of clinical training. Building a cadre of ophthalmology trainees well-versed in research during their medical training may foster an interest in research through the residency years, and beyond into academic and other research careers. In particular, instilling an interest in research early on may help encourage ophthalmologists to pursue additional research training after residency.^2^

In addition, opportunities to pursue ophthalmology research may also enhance engagement with the specialty. It is crucial to engage medical students in surgical specialties such as ophthalmology to continue to fill the pipeline into the specialty. Exposure to a surgical specialty early in medical school is a key differentiator in predicting matching into that specialty,^4^ and one recent survey of medical graduates found lack of exposure to ophthalmology as the #2 reason for not choosing this specialty.^5^ This lack of exposure is particularly acute for ophthalmology, which forms a shrinking portion of the core medical school curriculum in the United States.

With these benefits in mind, our institution developed a new summer research training program for first-year medical students interested in ophthalmology and received a T35 training grant from the United States National Eye Institute in 2020 to help support eight students in this program annually. There are currently ten such programs in the United States, only 4 of which are affiliated with Ophthalmology departments. Our program goals were to introduce students to basic and clinical research projects in ophthalmology and to provide exposure to clinical work in the discipline, with the hope of inspiring interest in the specialty and research more broadly. However, in 2020 the COVID-19 pandemic caused by the SARS-CoV2 novel coronavirus^6^ intervened after recruitment of students. Due to stay-at-home orders and institutional policy, we had to retool the program as virtual-only, while maintaining the overall goals.

The core component of our program was a research project with a faculty mentor. Evidence indicates that hands-on experience with research helps physicians be more critical consumers of research data, even when they do not continue with their own research. Surveys report that 50–90% of medical students completing a research elective had an "increased sense of confidence" in performing a literature search and critically appraising the literature.^7^^,^^8^ Given this, a research project was a crucial program element. However, the majority of our planned research projects were laboratory-based and therefore had to be replaced with at-home projects when campus laboratories were in "hibernation". Mentors and trainees rose to the challenge, devising projects including literature and systematic reviews, designing a survey study, developing an IRB protocol, and performing retrospective clinical studies. Trainees engaged with their mentors through one-on-one Zoom meetings and interacted with lab members through online lab meetings and individual discussions. An advantage of some of these projects compared to bench research was their rapid completion, raising the possibility of early publications for program students, which in turn will open doors to future research opportunities.

Beyond the research project, we set out to introduce our trainees to some discipline-specific content. Our medical school curriculum includes little ophthalmology content in the first year. Therefore we devised a vision science "boot camp" to introduce students to key concepts needed for their research, to excite them about the field, and to prepare them for upper-year ophthalmology electives. Topics included brief introductions to visual neuroanatomy, basic optics, imaging the eye, glaucoma, retinal disease, cornea, pediatric eye disease, and ophthalmic plastic and reconstructive surgery. These didactic sessions transitioned easily to a Zoom videoconference format. We also presented via Zoom a Responsible Conduct of Research session focused on the discussion of lessons learned from ethical transgressions from the ophthalmology field and/or how these could be avoided. As an added bonus, other trainees such as graduate students were able to attend these sessions in the virtual format.

To bridge the gap between research, didactics, and patient care, clinical shadowing for one half-day per week was a key component of our program. This had to be cancelled as medical students were temporarily restricted from clinical exposure during the early months of the COVID-19 pandemic. However, we were able to partially replace this with a curriculum of self-paced video lectures and clinical case studies. Attendance at virtual didactic sessions geared for our incoming residents likewise added to clinical topic exposure. Students also attended a school-wide series of career development seminars, again virtually, and department enrichment events such as Grand Rounds, Research Seminars, and Work in Progress seminars. A benefit of the virtual format was that it was easy for students to schedule attendance at these varied events, and for program directors to monitor attendance.

Finally, students prepared oral presentations of their research as part of the school-wide summer research program. An unexpected bonus of the virtual program was the feasibility of also showcasing these presentations as part of a virtual institution-wide Vision Research Day, giving the program trainees an extra opportunity to share their research-in-progress alongside PhD students and postdoctoral fellows. This was especially valuable during a season in which many external conferences were cancelled^9^ since it provided further exposure, presentation practice, and a reportable scholarly product for residency applications.

The COVID-19 pandemic upended all aspects of ophthalmology education,^10^^,^^11^ forcing creative use of online technologies.^12^^,^^13^ Since research opportunities for medical students are so important for building well-rounded physicians,^7^^, ^^8^ and for future career success,^14^ we developed an online summer research program. Our program complemented innovations made elsewhere in ophthalmology education and clinical skills training during the COVID-19 pandemic.^15^^,^^16^ "Virtualizing" a new summer research program necessitated compromises, notably the loss of bench research and clinical shadowing. However, the commitment of our clinical and research faculty, plus the trainees in the program, enabled program goals of exposure to ophthalmology clinical care and research projects to be met with surprising benefits of broadened scope, flexibility, and immediacy. Virtual research projects were devised and could be completed quickly while still building familiarity with research tools and methods. Virtual didactic sessions could be shared widely and more easily attended; these could potentially be made available to students beyond the institution in the future. Exposure to clinical cases through a variety of venues, although not a substitute for hands-on medical care, provided students with discipline-specific exposure. And virtual capstone research presentations allowed students to present in front of a larger (virtual) group than in-person posters would allow. Together, these findings offer a paradigm for maintaining medical student research programs in situations where in-person interaction is not possible.

### Acknowledgments

This program was supported by NIH/NEI T35EY031282.

### Conflict of Interest

The authors declare that they have no conflict of interest.
